# Exploring the effects of drug, disease, and protein dependencies on biomedical named entity recognition: A comparative analysis

**DOI:** 10.3389/fphar.2022.1020759

**Published:** 2022-12-21

**Authors:** Peifu Han, Xue Li, Xun Wang, Shuang Wang, Changnan Gao, Wenqi Chen

**Affiliations:** College of Computer Science and Technology, China University of Petroleum (East China), Qingdao, China

**Keywords:** biomedical named entity recognition, pre-training model, graph attention network, external knowledge, entity dependencies

## Abstract

**Background:** Biomedical named entity recognition is one of the important tasks of biomedical literature mining. With the development of natural language processing technology, many deep learning models are used to extract valuable information from the biomedical literature, which promotes the development of effective BioNER models. However, for specialized domains with diverse and complex contexts and a richer set of semantically related entity types (e.g., drug molecules, targets, pathways, etc., in the biomedical domain), whether the dependencies of these drugs, diseases, and targets can be helpful still needs to be explored.

**Method:** Providing additional dependency information beyond context, a method based on the graph attention network and BERT pre-training model named MKGAT is proposed to improve BioNER performance in the biomedical domain. To enhance BioNER by using external dependency knowledge, we integrate BERT-processed text embeddings and entity dependencies to construct better entity embedding representations for biomedical named entity recognition.

**Results:** The proposed method obtains competitive accuracy and higher efficiency than the state-of-the-art method on three datasets, namely, NCBI-disease corpus, BC2GM, and BC5CDR-chem, with a precision of 90.71%, 88.19%, and 95.71%, recall of 92.52%, 88.05%, and 95.62%, and F1-scores of 91.61%, 88.12%, and 95.66%, respectively, which performs better than existing methods.

**Conclusion:** Drug, disease, and protein dependencies can allow entities to be better represented in neural networks, thereby improving the performance of BioNER.

## 1 Introduction

The number of biomedical literature is increasing rapidly. On average, more than 3,000 new articles are published in peer-reviewed journals every day, excluding technical reports such as preprints and clinical trial reports in various archives. So far, PubMed (Roberts, 2001) has 33 million citations and abstracts in the biomedical literature. Reports containing valuable information about new discoveries and insights have been added to a large number of literature reports; meanwhile, the ever-increasing volume of biomedical literature has caused great challenges in extracting relevant and high-quality information. Therefore, there is an increasing demand for accurate biomedical text mining to extract information from the literature.

Named entity recognition (NER) is a fundamental task of natural language processing, which aims to identify named entities (NEs) in text, and is the basis for tasks such as network clustering (Su et al., 2021), disease module identification (Tian et al., 2020), relation extraction, knowledge graph construction, and link prediction (Su et al., 2020; Lei et al., 2021; Wang et al., 2021a; Wu et al., 2021; Cai et al., 2022). Benefiting from the use of neural network models (Ma and Hovy, 2016; Yang and Zhang, 2018) and pre-trained language models (LMs) (Akbik et al., 2018; Devlin et al., 2019), NER in natural language processing (NLP) has been extensively studied in general terms of NE types, such as person names, organizations, and departments, achieving human-level performance. The most representative model for NER is BiLSTM-CRF (Ma and Hovy, 2016; Tang et al., 2019), which utilizes a bidirectional long short-term memory (BiLSTM) network to encode biomedical texts and CRF to decode named entity labels. Ma and Hovy (2016) extended LSTM-CNNsCRF by utilizing a CNN and CRF. Zhang et al. (2018) used long short-term memory (LSTM) as a baseline model, combining multi-task learning and multi-step training to improve the performance of the clinical NER datasets. However, LSTM takes a long time to process long text; considering the context, it generally adopts a bidirectional structure (Kocaman and Talby, 2021), while BERT uses the attention mechanism, and the weight of each position of text relative to another position can be calculated in parallel, which is much faster than LSTM on the premise of sufficient computing resources. Other works replace the BiLSTM encoder with pre-trained language models, such as ELMo and BERT (Devlin et al., 2019), which consider deeper semantic features and farther contextual semantics, and those works obtain better experimental results. In addition, domain-specific pre-trained language models also bring improvement in clinical NER. In the past few years, the application of the recurrent neural network (RNN) (Li and Jiang, 2017; Ju et al., 2018; Hemati and Mehler, 20192019), convolutional neural network (CNN) (Korvigo et al., 2018; Zhu et al., 2018; Nie et al., 2021), and conditional random field (CRF) (Wang et al., 2018) in biomedical named entity recognition has promoted the development of biomedical text mining models. In recent years, in order to improve the performance of NER machine learning methods, external knowledge has been used as a complement to traditional contextual information, such as base information (Akbik et al., 2018), n-gram information (Li and Jiang, 2017), and part-of-speech (POS) tagging information (Devlin et al., 2019).

Biomedical named entity recognition (BioNER), which can help drug discovery (Wang et al., 2021b; Cai et al., 2022; Wang et al., 2022), is more challenging than it is in general fields. Researchers try to use various methods to improve the performance of BioNER, some introducing pre-trained models. Pre-trained transformer language models such as BERT (Devlin et al., 2019) and its variants such as RoBERTa (Liu et al., 2019) have brought significant performance gains on a variety of language tasks. BERT has been adopted in the biomedical domain. Lee et al. (2020) trained BERT on PubMed abstracts (PubMed) and PubMed central full-text articles (PMC) and proposed BioBERT for domain-specific language representation. Fang and Wang (2021) used a cased WordPiece vocabulary trained from a biomedical corpus, which also included all PubMed abstracts and 1 million PMC full-text articles, to promote Bioformer, which is a lighter model than BioBERT. BioNEs has more diverse context relations and richer semantic-related entities, such as drug molecules, targets, proteins, channels, and pathways. For example, “Alzheimer’s disease (AD) is a neurodegenerative disease characterized by progressive memory loss and dementia.” Here, there are four different descriptions of Alzheimer’s in this sentence which are “(AD),” “neurodegenerative disease,” “progressive memory loss,” and “dementia”—some of them are diseases, and some are symptoms. By observation, we find that if one of the words can be identified, then the other entities can be identified through dependencies. For instance, the disease entity “Alzheimer’s disease” can be identified through dependency “characterized by” and characteristic entity “progressive memory loss”. In addition, knowledge is also widely used in other text mining, while the one that contains the most dependencies is the knowledge graph. Some researchers have found that incorporating external knowledge can improve the performance of NER. The integration of external knowledge is used in deep learning models to improve their performances for NER. Wang et al. (2018) used distant supervision methods based on knowledge to improve the performances of clinical NER systems *via* injecting the representations of concepts I n KG into the representations of tokens. Wang et al. (2019) incorporated medical dictionaries into BiLSTM-CRF and achieved SOTA performance on the CCKS2017 dataset. Nie et al. (2021) used entities from the WikiData knowledge to promote NER models *via* concatenating concept embeddings and token embeddings. Xiong et al. (2022) integrated the boundary information on lexicon words from multiple knowledge graphs or knowledge graph(s) and lexicon(s). Chen et al. (2021) adopted both global co-reference relations and local dependency relations for building better entity mention representations for BioNER. However, most of them treated tokens in external knowledge equally also without considering the relationship between entities, which may be helpful for BioNER. Based on this consideration, we introduce a knowledge description by mining textual entity dependencies and expect to improve the NER performance. For example, the two entities in the knowledge graph, the diseases “Alzheimer’s disease” and “Senile Dementia,” belong to the “same as” entity dependence. If we can recognize that “Alzheimer’s disease” is a disease entity, we can more accurately infer whether the other word, “Senile Dementia,” is a disease entity according to the dependence.

In this study, we first perform word-level embeddings on biomedical domain text and knowledge graph entities using a BERT preprocessing model. Then, text word-level embeddings and entity dependencies are integrated using a graph neural network (GNN), specifically using graph attention (GAT) networks (Velickovic et al., 2018). GAT has shown good performance in many tasks, such as Chinese NER and short text classification (Hu et al., 2019; Sui et al., 2019). The experimental results show that the method proposed in this paper can obtain better word representation and further improve the recognition performance of biomedical named entities.

## 2 Materials and methods

**FIGURE 1:**
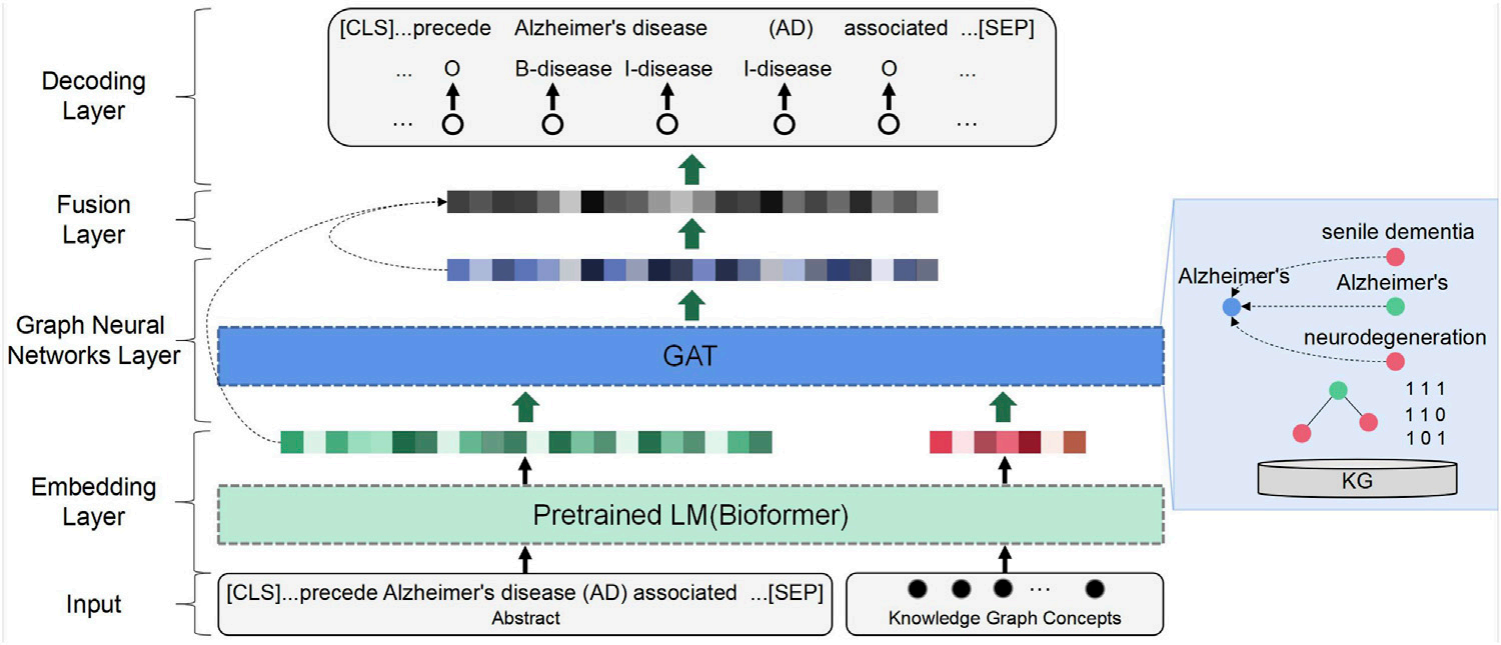
Overview structure of MKGAT.

MKGAT mainly consists of five layers, including an embedding layer, a graph neural network layer, a knowledge fusion layer, and a decoding layer (Figure 1). Sentences and nodes in the knowledge graph are fed into the pre-trained LM embedding model Bioformer to obtain semantic representation vectors. Then, the node representation incorporates local dependencies by considering adjacent node information and updates the node representation vectors by feeding them into a GAT network. The semantic feature vector and the updated node feature vector are fed into a multi-dimensional encoder, which generates a more coupled vector through feature blending. The decoder is then applied to this vector, and finally, the label indicates the entity category is the output.

### 2.1 External dependency knowledge

**FIGURE 2:**
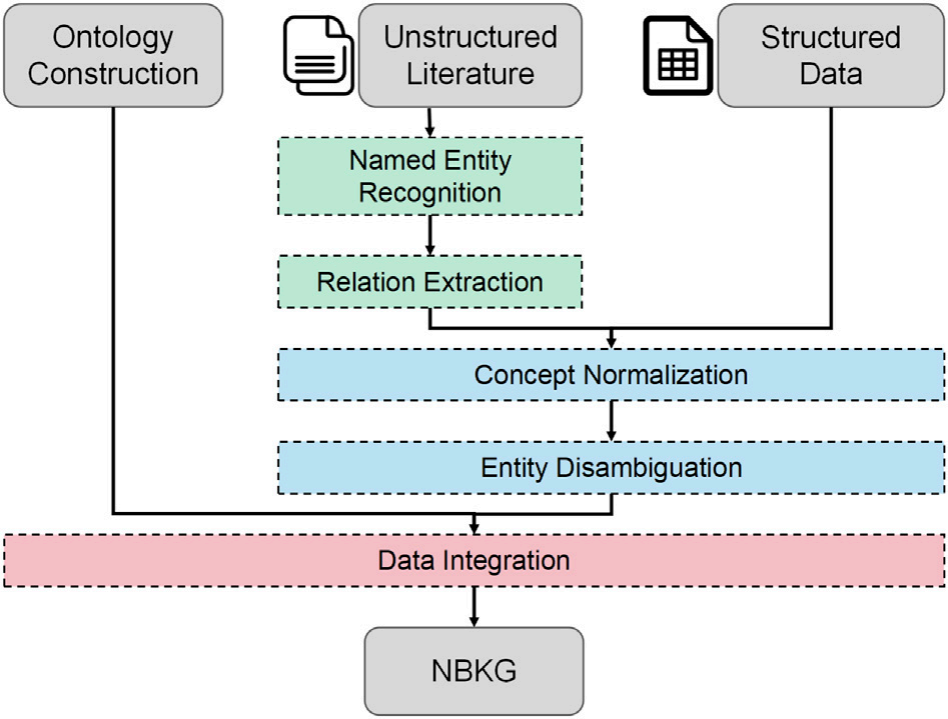
General process of NBKG construction.

To leverage dependency knowledge, we use a knowledge graph named the natural brain knowledge graph (NBKG) constructed by ourselves (Figure 2). We mine external text entity dependencies through the NBKG, hoping to improve the BioNER performance. The NBKG is developed based on a large number of medical, botany, and pharmacy encyclopedias, including PubMed (Roberts, 2001), BIOFACQUIM (Pilón-Jiménez et al., 2019), CMAUP (Zeng et al., 2018), and DrugBank (Wishart et al., 2018) databases, which contain 30,926 drug compounds, 21,771 targets, and over 100 kinds of diseases in three categories: “Brain Disease,” “Drug,” and “Target.” The types of edges in the NBKG include the same entity type dependency, such as “same as” and “belong to” and different entity type dependencies, such as “caused by” and “act on.” For example, the disease entity “Alzheimer’s disease” has the same entity type dependency “same as” with the disease entity “senile dementia,” and also, “Alzheimer’s disease” has a different entity type dependency “caused by” with the protein entity “Mfn2.” We match text words with the NBKG entity, and in this way, the external entity dependency for words in a sentence is imported to improve the representation accuracy of the whole-text entity space.

To build a domain knowledge graph, ontology needs to be used to define concepts and express relationships between concepts, to identify named entities and extract relationships from the unstructured literature, such as PubMed, to integrate knowledge with structured knowledge, such as DrugBank, and to disambiguate semantics. Finally, knowledge will be sorted into the form of a knowledge graph according to the concepts, attributes, and relationships defined by ontology; the general process is shown in Figure 2.

**FIGURE 3:**
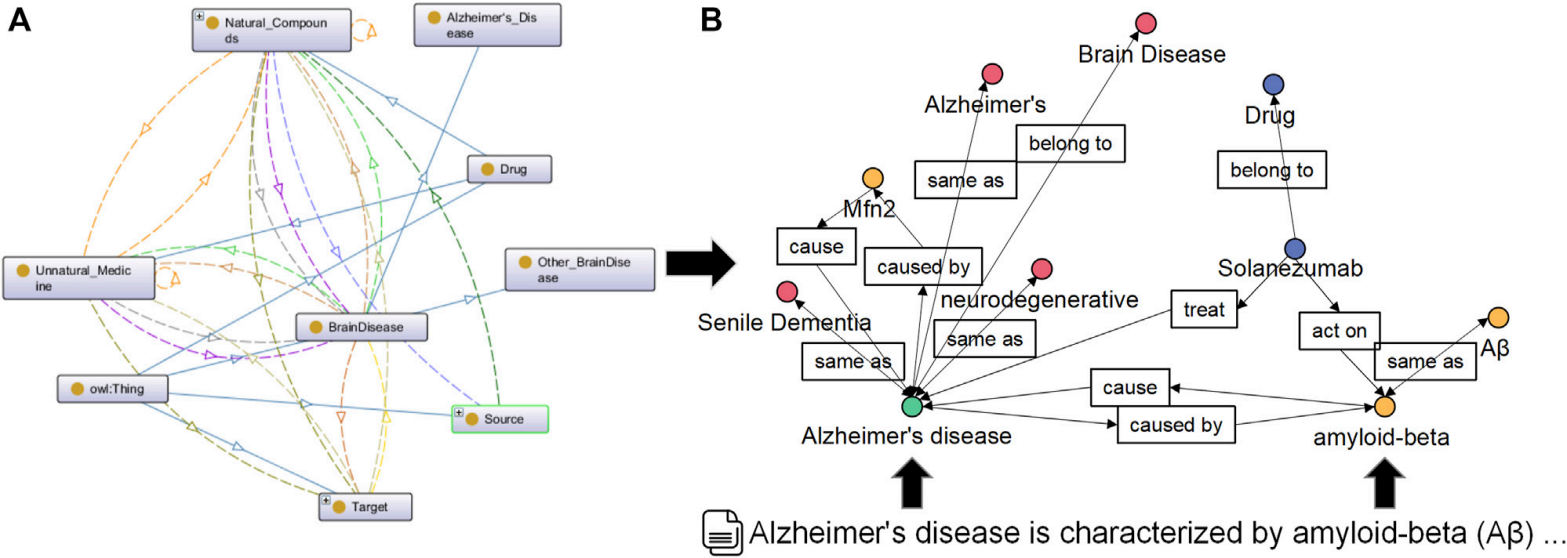
**(A)** Conceptual relationship in the NBKG ontology. **(B)** Overview structure of the NBKG.

Since the domain knowledge map is built, compared with extracting ontology structures from different databases, NBKG ontology is built manually, and the conceptual relationship displayed in the ontology is shown in Figure 3A. For unstructured knowledge, such as the PubMed literature, the finely tuned BioBERT is used to extract named entities and relationships. For entity normalization, we used the International Classification of Diseases, Tenth Edition (ICD-10), and other standards. The structure example of the final NBKG is shown in Figure 3B.

### 2.2 Model architecture

#### 2.2.1 Embedding layers

To obtain the context representation of each token of input sentence $X=\left\{x_{1},x_{2},\ldots ,x_{n}\right\}$ , we first apply Bioformer to the sentence as follows:

$$S=BERT\left(X\right),$$ (1)

where $S=\left\{s_{1},s_{2},\ldots ,s_{n}\right\}$ . The representation of each token of the knowledge graph node $V=\left\{v_{1},v_{2},\ldots ,v_{m}\right\}$ is first marked with special tags [CLS] and [SEP] for every word. Then, it is also initialized through Bioformer:

$$\begin{array}{l} \left[CLS\right]v_{i}\left[SEP\right]\\ V^{\prime }=BERT\left(V\right) \end{array},$$ (2)

where m is the number of nodes in the knowledge graph, $V^{\prime }=\left\{v_{1}^{\prime },v_{2}^{\prime },\ldots ,v_{m}^{\prime }\right\}$ .

#### 2.2.2 Graph neural network layer

The external entity dependency introduced by the knowledge graph is considered to enhance the context representation of the token. The computational process of GAT (Velickovic et al., 2018) can be summarized as follows, given a graph $G=\left(V,A\right)$ with nodes $v_{i}\in V$ and the adjacent matrix A . It is worth mentioning that we want to focus this exploration on comparing the influence of the presence or absence of external dependencies on the experiment rather than the influence of the type of relationship on the experiment, so we temporarily consider all external dependencies to be of the same type. The adjacent matrix A is defined according to the knowledge graph node $v_{i}$ and its adjacent node $v_{j}$ , in which $v_{i}$ also appears in the input sentence, where $A_{i,j}=1$ . At the same time, in order to facilitate feature fusion while maintaining the original semantic information on node entities that may appear in a sentence, the self-adjacency values of nodes are defined as $A_{i,i}=1$ .

The graph attention mechanism is used to aggregate the information on neighbor nodes and their corresponding standardized attention scores, and the attention score $\alpha_{i,j}^{k}$ of the k -th head is defined as follows:

$$\alpha_{i,j}^{k}=\frac{\exp\left(\sigma \left(a^{T}\left(W^{k}v_{i}^{\prime }\|W^{k}v_{j}^{\prime }\right)\right)\right)}{\sum_{z\in N_{i}}\exp\left(\sigma \left(a^{T}\left(W^{k}v_{i}^{\prime }\|W^{k}v_{z}^{\prime }\right)\right)\right)},$$ (3)

where $a^{T}$ is the learnable weight vector, $W^{k}$ is the parameter matrix of the k -th attention head of GNN, and z represents all the neighbors of node i . We compute $\alpha_{i,j}^{k}$ for nodes i and $j\in N_{i}$ , and $N_{i}$ is the first-order neighborhood of node i in the graph obtained from the adjacent matrix A . So, the node representation $g_{i}$ is updated to $h_{i}$ :

$$g_{i}=\sigma \left(\underset{j\in N_{i}}{\sum }\alpha_{i,j}^{k}W^{k}v_{j}^{\prime }+v_{i}^{\prime }\right)$$ (4)

$$h_{i}=concat\left(g_{i}\right)$$ (5)

$$H=\left(h_{1},h_{2},\ldots ,h_{n}\right)$$ (6)

where $\alpha_{i,j}^{k}$ is the attention score of the k -th head, σ represents the activation function LeakyReLU, and $v_{i}^{k}$ represents the graph node code $v_{i}^{\prime }$ under the k -th attention head, $k=\left\{1,2,\ldots ,K\right\}$ .

#### 2.2.3 Fusion layer

Similar to the method used by Chen et al. (2021), the sentence word embeddings S and GNN-updated node entity embeddings H are also fused using a fusion layer. The two kinds of embeddings are first projected into the same hidden space using a linear transformation and then added:

$$F=w_{1}S+w_{2}H,$$ (7)

where $w_{1}$ and $w_{2}$ are trainable weights, $F=\left\{h_{1}^{\prime },h_{2}^{\prime },\ldots ,h_{n}^{\prime }\right\}$ .

#### 2.2.4 Decoding layer

A conditional random field (CRF) is used for named entity label prediction. Given the input sentence $X=\left\{x_{1},x_{2},\ldots ,x_{n}\right\}$ with knowledge-enhanced representation $F=\left\{h_{1}^{\prime },h_{2}^{\prime },\ldots ,h_{n}^{\prime }\right\}$ , the probability of the label sequence $Y=\left\{y_{1},y_{2},\ldots ,y_{n}\right\}$ :

$$P\left(Y|X\right)=\frac{\exp\left(\sum_{i}W^{y_{i}}fh_{i}^{\prime }+T\left(y_{i-1},y_{i}\right)\right)}{\sum_{y\in D}\exp\left(\sum_{i}W^{y_{i}}fh_{i}^{\prime }+T\left(y_{i-1},y_{i}\right)\right)},$$ (8)

where D represents all possible label sequences of input X , $W^{y_{i}}$ represents the emission weight of $y_{i}$ for $h_{i}^{\prime }$ , and T represents the transition matrix weight of two adjacent labels. In addition, the negative log-likelihood function is used as the loss function as follows:

$$loss=-\overset{n}{\underset{i=1}{\sum }}\log\left(P\left(y_{i}|x_{i}\right)\right).$$ (9)

## 3 Experiments

### 3.1 Datasets

We tested the performance of the model on three general datasets in BioNER, NCBI-disease corpus (Dogan et al., 2014), BC2GM, and BC5CDR-chem (Li et al., 2016) datasets. The NCBI-disease corpus contains 793 PubMed abstracts, 6,892 disease mentions, and 790 unique disease concepts. BC2GM contains 20,703 labeled entities, and BC5CDR corpus consists of 1,500 PubMed articles with 4,409 annotated chemicals, which are used for the experiment. The NCBI-disease corpus is fully annotated at the mention and concept level to serve as a research resource for the biomedical natural language processing community. BC2GM is from a gene mention tagging task, as part of the BioCreative II challenge, which is concerned with the named entity extraction of gene and gene product mentions in the text. BC5CDR is introduced in BioCreative V CDR task corpus, which is a resource for chemical disease relation extraction.

The number of common disease concepts existing both in NBKG and NCBI-disease is 92, and the number of common gene/protein concepts existing both in NBKG and BC2GM is 1,468, which is displayed in the “common” column in the table, while the number of common gene/protein concepts existing both in NBKG and BC5CDR-chem is 257 (Table 1).

**TABLE 1:** Quantity of entities in the NBKG and three corpora.

	NCBI-disease corpus	BC2GM	NBKG	Common
Disease	3,924	—	128	92
Gene/protein	—	20,703	21,771	1,468
Drug	4,409	—	2,499	257

### 3.2 Experimental settings

We use the “BIO” (B—begin, I—inside, and O—outside) labels to represent the boundaries of entity mentions. We set the maximum length of sentences to 175 for NCBI-disease corpus, 300 for BC2GM, and 200 for BC5CDR-chem, which covers over 99% of sentences. The hidden size of the BERT is set to 512. The dropout rate is set to 0.2 in BERT and the GNN updating layer. The Adam optimization algorithm is used as the optimizer with a learning rate of 1e-5. We use a layer GAT to model external knowledge with 50 hidden nodes. The number of epochs we set is 10, and the batch size is set to 35 at each epoch. Bioformer, pre-trained from the scratch on the same corpus as the vocabulary, which included 33 million PubMed abstracts and 1 million PMC full-text articles, is used to embed the sentence. We train the entities embedding on the NBKG using Bioformer. All hyper-parameters in Bioformer for entities embedding training are set to their default values except the following hyperparameters: the number of epochs is set to 10, and the batch size is set to 35. All experiments are conducted on RTX 3090.

### 3.3 Evaluation metrics

We use several metrics to evaluate the model performance, including precision, recall, and F_1_ score. Precision is the ratio of true positives in the identified positive samples, which reflects the classification accuracy of the model for BioNEs tokens:

$$Precision=\frac{TP}{TP+FP},$$ (10)

where TP indicates the number of positive classes predicted as positive classes, and FP indicates the number of negative classes predicted as positive classes. Recall represents the proportion of all positive samples in the test set that are correctly identified as positive samples, which reflects the ability of the model to distinguish BioNEs:

$$Recall=\frac{TP}{TP+FN},$$ (11)

where FN indicates the number of positive classes predicted as negative classes. The F_1_ score is one of the most commonly used metrics in classification and information retrieval, reflecting the average performance of model precision and recall:

$$F_{1}=\frac{2*Precision*Recall}{Precison+Recall}.$$ (12)

## 4 Results

### 4.1 Comparison with existing methods

Using 3,924 disease BioNEs, 20,703 gene/protein BioNEs and 21,899 knowledge graph tokens, we make a comparison of our model with existing methods on the two datasets (Table 1).

Layered-BiLSTM-CRF without considering external knowledge was used for the test first. Then we compare our method with the SOTA method using BERT as the embedded layer, including BERT base, BioBERT v1.0, BioBERT v1.1, and MKRGCN considering external knowledge.

Layered-BiLSTM-CRF is a neural layered model for nested NER.

MTM-CW is a multi-task learning model which shares character-level embedding parameters, word-level embedding parameters, and character and word layer embedding parameters for NER.

BERT-base (Devlin et al., 2019) is a contextualized word representation model that is based on a masked language model and pre-trained using bidirectional transformers. We use the embeddings from it with BiLSTM-CRF architecture as a baseline.

BioBERT v1.0 (Lee et al., 2020) is a BERT-base pre-trained with 200 thousand PubMed abstracts and 270 thousand PMC full-text articles. We use the NER fine-tuning version of it with BiLSTM-CRF architecture as a baseline.

BioBERT v1.1 (Lee et al., 2020) is the same as BioBERT v1.0 but pre-trained with 1 million PubMed abstracts.

MKRGCN (Xiong et al., 2022) leverages lexicon of words in Chinese and domain knowledge graph concepts to consider the boundaries of NEs.

EnRel-G (Chen et al., 2021b) incorporates entity mention relations based on both global co-reference relations and local dependency relations by graph neural networks.

We run our model five times with different seeds, and report the precision, recall, and F_1_ score.

**FIGURE 4:**
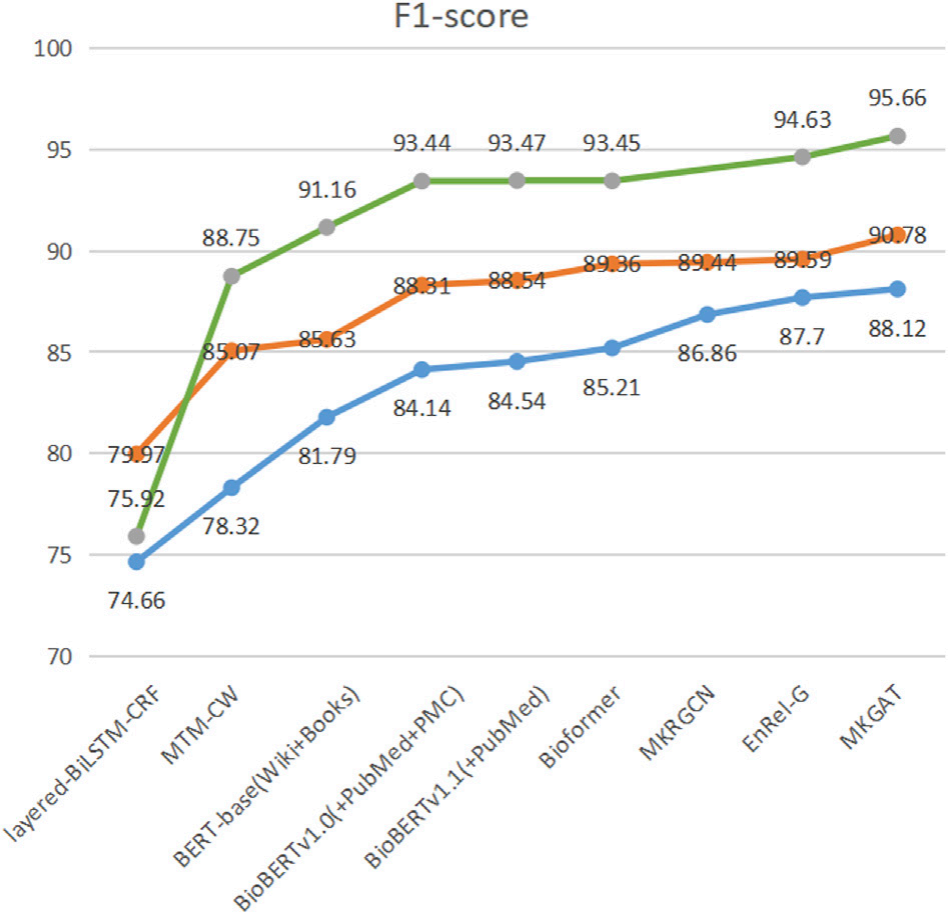
F1-score of MKGAT on three datasets.

We have achieved the best results, as highlighted. MKGAT outperforms layered-BiLSTM-CRF, MTM-CW, BERT-base (Wiki + Books), BioBERT v1.0 (+PubMed + PMC), BioBERT v1.1 (+PubMed), and MKRGCN on the NCBI-disease corpus, BC2GM and BC5CDR-Chem datasets (Table 2). MKGAT achieves an F_1_ score of 90.78% on the NCBI-disease dataset, an F_1_ score of 88.12% on the BC2GM dataset, and an F_1_ score of 95.66% on the BC5CDR-chem dataset, higher than the existing best performances reported by Xiong et al. (2022) by 0.98% in F_1_ score on the NCBI-disease dataset and 0.31% in F_1_ score on the BC2GM dataset, respectively. Compared with Bioformer, MKGAT achieves higher F_1_ score by 1.42%, 2.91%, and 2.21% on the NCBI-disease corpus, BC2GM, and BC5CDR-chem datasets, respectively. The improvements from external knowledge encoding and the fusion layer are significant. In the case of the methods that can utilize KG concepts and local dependency, using them together may be more effective. For example, MKGAT performs better than MKRGCN on the NCBI-disease corpus but sometimes worse on the BC2GM (MKRGCN-max is higher than MKGAT-min). This result indicates that leveraging local dependency from external knowledge for NER is not simple. The reason may be that the representation space of article words is different from that of KG concepts. We can see that MKGAT performs better on NCBI-disease corpus than BC2GM as same as other models (Figure 4). This may be caused by the scale of the dataset and the length of the entities in them. MKGAT provides an effective attention mechanism to leverage local dependency for NER and also shows potential to be applied to other tasks.

**TABLE 2:** Comparison of different methods on the NCBI-disease corpus, BC2GM and BC5CDR-Chem datasets.

Method	Datasets								
	NCBI-disease corpus			BC2GM			BC5CDR-chem		
	Precision	Recall	F_1_	Precision	Recall	F_1_	Precision	Recall	F_1_
Layered-BiLSTM-CRF	83.63	76.26	79.97	77.42	72.10	74.66	80.20	65.19	75.92
MTM-CW	85.43	84.71	85.07	79.43	77.25	78.32	88.98	88.54	88.75
BERT-base (Wiki + Books)	84.12	87.19	85.63	81.17	82.42	81.79	90.94	91.38	91.16
BioBERT v1.0 (+PubMed + PMC)	87.78	88.85	88.31	85.04	83.26	84.14	93.27	93.61	93.44
BioBERT v1.1 (+PubMed)	87.24	89.89	88.54	84.13	84.95	84.54	93.68	93.26	93.47
Bioformer	88.01	90.76	89.36	85.05	85.38	85.21	93.52	93.39	93.45
MKRGCN Xiong et al. (2022)	—	—	89.44 (±0.36)	—	—	86.86 (±0.40)	—	—	—
EnRel-G Chen et al. (2021)	89.06	90.12	89.59	87.82	87.59	87.70	94.69	94.58	94.63
MKGAT	**90.04 (±0.12)**	**91.52 (±0.63)**	**90.78 (±0.36)**	**88.19 (±0.72)**	**88.05 (±1.19)**	88.12 (±0.95)	**95.71 (±0.35)**	**95.62 (±0.74)**	**95.66 (±0.56)**

### 4.2 Ablation study

We conduct an ablation study on the three BioNER datasets to evaluate the performance of the embedding mode and knowledge from KG on the MKGAT. The models with “concept” are the models using BERT to embed knowledge graph concepts without relation, and the models with “relation” are the models using the GNN to model knowledge graph concepts and their relations (Table 3). It is worth mentioning that the effect of the structure of BiLSTEM + CRF is compared. The results show that replacing the BiLSTM module with a full connection can still achieve better results, which may be related to our concern about the relationship from additional knowledge rather than context. Comparing the results of Bioformer with those of the Bioformer concept, we can see both the embedding mode and source of knowledge can bring improvements. The performance that Bioformer + GAT relation gets better scores on precision, recall, and F_1_ shows that considering relations of concepts can help find out a better representation space for all words in articles so as to identify entities more accurately.

**TABLE 3:** Ablation experiments for different embeddings and network structures.

Method	NCBI-disease corpus			BC2GM			BC5CDR-chem		
	Precision	Recall	F_1_	Precision	Recall	F_1_	Precision	Recall	F_1_
BiLSTM + GAT-relation	82.22	75.63	78.79	71.52	65.74	68.51	83.12	83.28	83.20
BioBERT v1.0 + GAT relation	88.64	89.38	89.01	86.81	87.06	86.93	90.66	90.52	90.59
BioBERT v1.1 + GAT relation	89.53	90.87	90.20	87.49	87.73	87.61	92.91	92.83	92.87
Bioformer	88.01	90.76	89.36	85.05	85.38	85.21	92.87	92.68	92.77
Bioformer concept	88.35	90.73	89.52	85.73	85.91	85.82	93.01	93.14	93.07
Bioformer + RGCN relation	88.91	90.16	89.53	87.68	87.01	87.34	93.45	93.79	93.62
Bioformer + GAT relation	**90.71**	**92.52**	**91.61**	**88.19**	**88.05**	**88.12**	**95.71**	**95.62**	**95.66**

### 4.3 Fine-grained study

We conduct a fine-grained study on the two NER datasets to identify which kind of entity can be recognized effectively in MKGAT.

**FIGURE 5:**
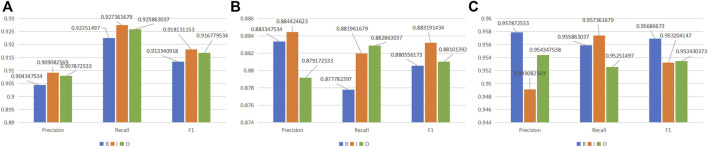
MKGAT performance for label “BIO” on **(A)** NCBI-disease, **(B)** BC2GM, and **(C)** BC5CDR-chem datasets.

It is shown that MKGAT achieves an F1 score of 90.33%, 91.81%, and 91.67% for labels “B,” “I,” and “O,” respectively, on the NCBI-disease corpus (Figure 5A), an F1 score of 88.05%, 88.32%, and 88.10% for labels “B,” “I”, and “O,” respectively, on the BC2GM (Figure 5B), and an F1 score of 95.69%, 95.32%, and 95.34% for labels “B,” “I,” and “O,” respectively, on the BC5CDR-chem dataset (Figure 5C). Compared with “B” and “O,” MKGAT achieved a higher score on label “I”. The reason may be that words with label “I” can be better characterized because of their greater quantity.

## 5 Discussion

From a pharmacological point of view, medical entities such as drugs, diseases, targets, and pathways are inseparable, and any of them will have an impact on the action of the drug. Then, for related research work in biomedicine, it is necessary to consider the dependencies between them, including BioNER. With the advent of the Internet era, especially with the outbreak of COVID-19, a wealth of information about diseases, drugs, and receptors has accumulated. However, most of the information is stored in the form of the literature. In addition to unstructured data, such as text data, many related semi-structured and unstructured data in different forms have also emerged, such as knowledge graphs. Therefore, it seems to us most natural to carry out biomedical named entity identification in the context of incorporating external knowledge. In this paper, we use a simple knowledge graph constructed by ourselves from PubMed (Roberts, 2001), BIOFACQUIM (Pilón-Jiménez et al., 2019), CMAUP (Zeng et al., 2018), and DrugBank (Wishart et al., 2018) databases. In order to take advantage of the dependencies in the knowledge graph, we match the entities in the graph with the words in the training sentences. To explore the effects of drug, disease, and target dependencies on BioNER, we constructed a model which first performs word-level embeddings on biomedical domain text and knowledge graph entities using a BERT preprocessing model. Then, text word-level embeddings and entity dependencies are integrated by using a graph neural network (GNN), specifically using graph attention (GAT) networks (Velickovic et al., 2018). GAT has shown good performance in many tasks, such as Chinese NER and short text classification (Hu et al., 2019; Sui et al., 2019).

To test the effect of the model, we conduct experiments using real-world datasets and compare them with seven currently more advanced methods (Table 2). MKGAT achieves an F1 score of 90.78% on the NCBI-disease dataset and an F1 score of 88.12% on the BC2GM dataset, higher than the existing best performances reported by Xiong et al. (2022) (Fang and Wang, 2021) by 0.98% in F1 score on the NCBI-disease corpus dataset and 0.31% in F1 score on the BC2GM dataset, respectively. Compared with Bioformer, MKGAT achieves higher F1 scores by 1.42% and 2.91% on the NCBI-disease dataset and the BC2GM dataset, respectively. The improvements from external knowledge encoding and the fusion layer are significant. Compared with MKRGCN (Xiong et al., 2022) of “integrating entity-related words with external knowledge,” our model is also very effective, indicating that “integrating entity dependencies” can also improve the accuracy of biomedical entity recognition. Compared with EnRel-G (Chen et al. (2021)), which incorporates entity-mentioned relations based on both global co-reference relations and local dependency relations, our model is more accurate, which means external knowledge brings more effective information. In addition, there are LSTM-based methods that have achieved good results in recent years, such as the SparkNLP’s BiLSTM-CNN structure model (Kocaman and Talby, 2021). However, because LSTM cannot perform parallel computing, this method is less efficient than the model proposed in this paper, so no further discussion will be made.

We also observed the performance of MKGAT using different modules on the three datasets (Table 3). It is found that when we do not use the pre-training model to fine-tune and only use the RNN to focus on the semantics within sentences, the performance degradation is serious, which confirms that compared with ordinary NEs, the semantic relationship of BioNEs is much more complex. The recognition accuracy is also unsatisfactory when we do not consider the data in the knowledge graph. This result confirms the effectiveness of our choice to incorporate external dependency knowledge in the process of identifying entities.

So is it really a dependency that affects the accuracy of the identified entity? We thought of this question and explored it. First, we only use the “entities in the knowledge graph corresponding to the words in the training set” to test. Then, we compare it with the test results using the “relational entities corresponding to the entities in the knowledge graph,” and we find that the results of integrating the relationship are better. However, it is worth noting that the improvement brought by dependencies is not much from the results alone. Why is the dependency relationship not improved so much? Later, we thought about whether the semantic relationship of BioNEs in texts is complicated, which leads to the confusion of recognition after incorporating the external dependency relationship. Therefore, we take the dependency entity as an attribute of BioNEs through GAT (Velickovic et al., 2018) and try to make the model focus on BioNEs themselves. The results show that it is helpful. Due to the complexity of the structure of BioNEs, such as “ (), numbers, etc.”, in order to explore the completeness of the model’s recognition, we also compared the boundary and internal recognition of BioNEs on three datasets (Figure 5). We found that although MKGAT can accurately identify NEs, the identification accuracy of boundaries is not as high as that of the interior, which also explains the direction of future research for us.

Although the model presented in this paper provides valuable insights into the application of pharmacological dependencies on BioNER, it is also important to consider the limitations to this study. As mentioned previously, it can be seen from the results that MKGAT is not accurate enough to identify the boundary of the entity; from the perspective of external knowledge, the knowledge graph constructed by us cannot cover all possible entities involved, resulting in the inaccurate recognition of individual BioNEs, and from the way of external knowledge integration, all kinds of local dependencies of entities in the knowledge graph are represented as an adjacent matrix, which may lose some information. It is important to remember, therefore, that the model presented here simply provides an idea for BioNER to leverage external knowledge.

## Data Availability

The original contributions presented in the study are included in the article/Supplementary Material; further inquiries can be directed to the corresponding author.
